# Depression and Suicide Literacy among Canadian Sexual and Gender Minorities

**DOI:** 10.1080/13811118.2020.1769783

**Published:** 2020-06-12

**Authors:** Olivier Ferlatte, Travis Salway, John L. Oliffe, Simon M. Rice, Mark Gilbert, Ingrid Young, Lisa McDaid, John S. Ogrodniczuk, Rod Knight

**Keywords:** Depression, gender identity, literacy, sexual and gender minorities, sexual orientation, suicide

## Abstract

The purpose of this study was to examine and compare depression and suicide literacy among Canadian sexual and gender minorities (SGM). Online surveys comprised of the 22-item depression literacy scale (D-LIT) and the 12-item literacy of suicide scale (LOSS) were completed by 2,778 individuals identifying as SGM. Relationships between depression and suicide literacy and demographic characteristics were evaluated using multivariable linear regression. Overall, SGM correctly answered 71.3% of the questions from the D-LIT and 76.5% of the LOSS. D-LIT scores were significantly lower among cisgender men and D-LIT and LOSS scores were lower among transgender women when compared to cisgender women. LOSS and D-LIT scores were significantly lower among SGM without a university degree (compared to those with a university degree) and among SGM from ethnic minority groups (compared to White SGM). D-LIT scores, but not LOSS scores, were significantly lower among Indigenous SGM compared to White SGM. The findings provide evidence of differences in suicide and depression literacy between SGM subgroups along multiple social axes. Interventions to increase depression and suicide literacy should be prioritized as part of a mental health promotion strategy for SGM, targeting subgroups with lower literacy levels, including cisgender men, transgender women, Indigenous people, racialized minorities, and those without a university degree.

## INTRODUCTION

High rates of depression and suicide among sexual and gender minorities (SGM)^1^ have motivated an array of studies aimed at detailing these epidemics and their specific risk factors (Haas et al., 2011; Hottes, Bogaert, Rhodes, Brennan, & Gesink, 2016; King et al., 2008; Marshall, Claes, Bouman, Witcomb, & Arcelus, 2016; Plöderl et al., 2013). Consistent with international studies (Ross et al., 2018), a Canadian population probability survey found a prevalence of self-reported mood disorders (including depression) among sexual minorities between 15–25% (Pakula, Shoveller, Ratner, & Carpiano, 2016), while a study of transgender people in Ontario, Canada, indicated that 66.4% of participants had symptoms consistent with depression (Rotondi et al., 2011). These trends are worrisome, as it is well established that, if untreated, depression increases suicide risk (Hawton, Casañas i Comabella, Haw, & Saunders, 2013). A systematic review estimated the prevalence of lifetime suicide attempts among sexual minorities to be 11–20% (Hottes et al., 2016), while a respondent-driven study of transgender individuals in Ontario found that 22–43% of transgender individuals had attempted suicide (Rotondi et al., 2011). Within the literature in this area, stigma and minority stress have emerged as the primary explanations for these elevated rates of depression and suicide among SGM (Haas et al., 2011; Plöderl et al., 2013; White Hughto, Reisner, & Pachankis, 2015).

In spite of the large burdens of depression and suicide among SGM, few SGM-specific mental health resources are available in Canada (Veltman & Chaimowitz, 2014), most require out-of-pocket payment, and the few that are free are volunteer-run and have long waitlists. Furthermore, despite high mental health service utilization among SGM (Tjepkema, 2008), many SGM report unmet mental health care needs (Salway, Ferlatte, et al., 2019; Simeonov, Steele, Anderson, & Ross, 2015). Limits to SGM-tailored resources are compounded by a general scarcity in accessible and affordable mental health services; among Canadians, fewer than half of the population with depression receive healthcare, with wide variation by depression severity, age, education, and other social characteristics (Salway, Ferlatte, et al., 2019; Simeonov et al., 2015). This is concerning not only because of the relationship between depression and suicide but also due to the often close relationships between untreated depression among SGM and other health issues, such as substance use disorders and sexual health–related outcomes (e.g., sexually transmitted infections; Ferlatte, Dulai, Hottes, Trussler, & Marchand, 2015).

A major challenge to improving timely access to mental health services is the lack of awareness of signs and symptoms of mental illnesses, and how these illnesses can be treated and/or managed (Jorm, 2012; Jorm et al., 2006). Indeed, mental health literacy has been described as a critical determinant of mental health, as well as a prerequisite to effective mental health promotion (Jorm, 2012; Jorm et al., 1997). Mental health literacy is defined as “knowledge and beliefs about mental disorders which aid their recognition, management or prevention”(Jorm et al., 1997). In the context of depression and suicide, literacy refers to the individual’s ability to recognize depression and suicide symptoms and risk factors and to make informed decisions about treatment (Batterham, Calear, & Christensen, 2013; Wang et al., 2007). Low depression literacy has been found to impede engagement with health professionals (Bonabi et al., 2016; Cheng, Wang, McDermott, Kridel, & Rislin, 2018; Ho et al., 2018; Tomczyk et al., 2018), and emerging evidence is showing that lower suicide literacy is associated with greater stigmatizing attitudes toward suicide (Batterham et al., 2013) and that individuals with high suicide literacy have more positive attitudes toward help-seeking for suicide ideation (Calear, Batterham, & Christensen, 2014).

Despite the burden of depression and suicide among SGM, there is a paucity of research that examines depression and suicide literacy in this diverse population. A search within the main health and psychology research databases (Pubmed and PsychInfo) yielded a single article that explicitly explored mental health literacy among SGM. The study, conducted in Switzerland, measured depression literacy by presenting a depression case vignette to 762 gay men, of whom only 44.8% correctly identified the case as depression (Wang, Häusermann, & Weiss, 2014). The study found that current and past experience of depression increased the likelihood of identifying the vignette correctly (Wang et al., 2014).

The lack of depression and suicide literacy studies among SGM challenges the effective development and evaluation of targeted interventions, particularly in the context where practitioners and health promoters often assume that SGM have high health literacy levels (Gilbert, Dulai, Wexel, & Ferlatte et al., 2015). As such, the purpose of the present study is to examine and compare depression and suicide literacy among Canadian SGM, including differences according to gender, sexual identity, and other demographic characteristics.

## MATERIALS AND METHODS

To measure depression and suicide literacy among Canadian SGM, an online survey was developed by a group of SGM and mental health researchers in collaboration with mental health professionals, public health professionals, and SGM community partners. The survey, which was anonymous, was available on the principal investigators’ institutional survey platform (Fluidsurvey^tm^). The survey was promoted on social media (e.g., Facebook, Twitter) and through Canadian SGM community groups’ e-mail distribution lists, websites, and social media sites. Respondents were eligible to participate if they self-identified as a sexual minority (gay, lesbian, bisexual, two-spirit, queer, pansexual, asexual) and/or gender minority (transgender, genderqueer, gender nonbinary), resided in Canada, were at least 18 years of age, and were able to complete the questionnaire in French or English (Canada’s official languages). Data were collected from November 2–December 21, 2017.

### Measures

Depression literacy was measured using the depression literacy scale (D-LIT), the most widely used scale to measure depression literacy (Wei, McGrath, Hayden, & Kutcher, 2015), which has been previously validated among the general population (Griffiths, Christensen, Jorm, Evans, & Groves, 2004; Gulliver et al., 2012). The D-LIT has an internal consistency of *α* = .70 (Gulliver et al., 2012). It is comprised of 22 statements with three response options—true, false, and do not know—measuring knowledge related to risk factors, symptoms, and treatment options. Respondents’ depression literacy scores were based on the number of correct answers (scores ranging from 0–22), with higher scores indicating greater depression literacy and lower scores indicating poorer literacy.

Suicide literacy was assessed using the short form of the only validated tool to measure suicide literacy, the literacy of suicide scale (LOSS; Batterham et al., 2013). The LOSS was not validated using classical test theory; rather, item response theory was used to identify items that had the strongest discrimination of the underlying literacy construct (Calear et al., 2014). The LOSS comprised 12 items measuring knowledge related to suicide risk factors, signs, and treatment options. For each statement, respondents answered true, false, or do not know. Suicide literacy was evaluated based on the number of correct answers (scores ranging between 1–12), with a higher score indicating greater suicide literacy. Because of the research team’s interest in SGM suicide, an additional item was added to measure respondents’ knowledge of the elevated risk of suicide in the SGM community (Bauer, Scheim, Pyne, Travers, & Hammond, 2015; Haas et al., 2011; Hottes et al., 2016). This item was not included in the tabulation of the suicide literacy score.

Demographic characteristics were selected based on prior literature identifying intersecting social positions related to mental health among SGM (Bostwick et al., 2014; Ferlatte et al., 2018; Salway, Ross, et al., 2019; Steele et al., 2017) and included gender, sexual orientation, age, education, income, and ethnicity. Gender was measured using a two-step approach combining sex at birth and gender identity (Bauer, Braimoh, Scheim, & Dharma, 2017), which resulted in six gender categories: cisgender men, cisgender women, transgender men, transgender women, gender nonbinary, and other (e.g., two-spirit). Sexual orientation included gay/lesbian, bisexual, queer, asexual, pansexual, straight, and other (because of low cell count, straight was collapsed with other but this group was not included in the analysis due to the diversity of identities represented). Respondent age was aggregated to five categories: under 20, 20–29, 30–39, 40–49, 50 + years. For education, respondents reported their highest educational attainment: high school or less, college or some university, and completed university. Respondents were asked as a categorical variable to report their individual income before taxes and deductions in the past 12 months. Income was examined using three groups: under CAD$20,000 (representing individuals under the low-income cutoff for single Canadians), CAD$20,000–49,999 (representing individuals above the low-income cutoff but bellow the Canadian average), and CAD$50,000 or more (representing individuals with an income above the Canadian average; Statistics Canada, 2020a, 2020b). Respondents reported their ethnicity and three groups were created: White, Indigenous (Indigenous, Aboriginal, First Nations, and Métis), and ethnic minorities (e.g., Black, Asian, Latino, or Middle Eastern).

### Data analysis

Once the survey was closed, the data were imported to SPPS version 23 and duplicates were removed prior to conducting analysis. First, the mean score of the D-LIT and LOSS scales were calculated separately. Then, to explore multivariable relationships between depression and suicide literacy and demographic characteristics (explanatory variables), multiple linear regressions were used with the D-LIT and LOSS scores as dependent variables. Coefficients with *p* < 0.05 were considered statistically significant. We checked for multicollinearity issues using variance inflation factors (VIF); all VIFs were smaller than three.

### Ethics

The research protocol was reviewed and approved by the Behavioural Research Ethics Board of the University of British Columbia (ref #H17-01592). Survey respondents provided informed consent before completing the online questionnaire. Participation was voluntary and respondents could elect not to answer any question or stop their participation at any time.

## RESULTS

A total of 2,778 Canadian SGM completed the online survey and were included in the present analysis. A detailed description of the demographic characteristics of the sample is available in Table 1. Overall, 71.6% of the responses to the D-LIT questionnaire (mean score 15.68, *SE* .056, range 22, interquartile range 4) and 76.5% of the responses to the LOSS questionnaire were answered correctly by respondents (mean score 8.06, *SE* .035, range 12, interquartile range 2; see Tables 2 and 3). Fewer than 40% of respondents correctly answered the following depression literacy items: “Clinical psychologists can prescribe antidepressants” (false; 38.4% correct); “Reckless and foolhardy behavior is a common sign of depression” (false; 33.2%); “Many treatments for depression are more effective than antidepressants” (false; 17.2%), and “Counseling is as effective as cognitive behavioral therapy” (false; 14.5%). By contrast, more than 95% of respondents were able to recognize depressive symptoms such as “Loss of confidence and poor self-esteem may be a symptom of depression” (true; 98.8%), “Eating too much or losing interest in food may be a sign of depression” (true; 98.5%), “Sleeping too much or too little may be a sign of depression” (true; 98.2%), and “People with depression may feel guilty when they are not at fault” (true; 95.9%).

**TABLE 1.** Demographic characteristics of participants of a Canadian survey about sexual and gender minorities mental health (*N* = 2,778).

Demographic variables	*n* (%)
Gender	
Cisgender men	623 (22.4)
Cisgender women	1,157 (41.6)
Transgender men	209 (7.5)
Transgender women	82 (3.0)
Nonbinary	561 (20.2)
Other	146 (5.3)
Sexual orientation	
Gay/Lesbian	1,032 (37.1)
Bisexual	615 (22.1)
Queer	486 (17.5)
Asexual	133 (4.6)
Pansexual	369 (13.3)
Other (including straight)	143 (5.1)
Age	
Under 20	636 (22.9)
20–29	1245 (44.9)
30–39	449 (16.2)
40–49	176 (6.3)
50+	267 (9.6)
Education	
Some high school	
Completed high school	473 (17.0)
Some college or university	1,383 (49.8)
University degree	907 (32.6)
Other/prefer not to say	15 (0.5)
Individual Income	
Under 20,000	1,500 (54.0)
20,000–49,999	714 (25.7)
50,000 or more	382 (13.8)
Prefer not to say	182 (6.6)
Ethnicity	
White	2,147 (77.3)
Indigenous	283 (10.2)
Asian	157 (5.7)
Black	46 (1.7)
Middle-Eastern	37 (1.3)
Latino	58 (2.1)
Other	42 (1.5)
Prefer not to say	16 (0.6)

**TABLE 2.** Canadian sexual and gender minorities responses to the Depression Literacy Scale (D-lit).

Statement	Selected	Selected	Selected
	True	False	Don’t know
Loss of confidence and poor self-esteem may be a symptom of depression.	98.8	0.4	0.7
Eating too much or losing interest in food may be a sign of depression.	98.5	0.5	1.0
Sleeping too much or too little may be a sign of depression.	98.2	0.9	0.9
People with depression may feel guilty when they are not at fault.	95.9	1.4	2.7
Many famous people have suffered from depression.	94.8	0.7	4.5
Depression does not affect your memory and concentration.	2.8	92.8	4.4
Most people with depression need to be hospitalized.	2.1	90.9	7.1
Antidepressant medications usually work straight away.	1.2	90.8	8.0
People with depression should stop taking antidepressants as soon as they feel better.	1.6	90.1	8.2
People may move more slowly or become agitated as a result of their depression.	86.0	4.2	9.8
People with depression often hear voices that are not there.	5.2	82.8	12.1
Having several distinct personalities may be a sign of depression.	6.3	78.1	15.6
Not stepping on cracks in the footpath may be a sign of depression.	4.5	74.7	20.7
Moderate depression disrupts a person’s life as much as multiple sclerosis or deafness.	70.9	10.4	18.7
Of all the alternative and lifestyle treatments for depression, vitamins are likely to be the most helpful.	5.8	63.2	31.0
Cognitive behavioral therapy is as effective as antidepressants for mild to moderate depression.	53.9	4.9	41.2
People with depression often speak in a rambling and disjointed way.	24.6	52.6	22.9
Antidepressants are addictive.	21.5	51.9	26.6
Clinical psychologists can prescribe antidepressants.	35.3	38.4	26.3
Reckless and foolhardy behavior is a common sign of depression.	45.6	33.2	21.2
Many treatments for depression are more effective than antidepressants.	36.2	17.2	46.6
Counselling is as effective as cognitive behavioral therapy for depression.	40.6	14.5	45.0
TOTAL OF CORRECT ANSWER	71.27		
TOTAL OF INCORRECT ANSWER	11.67		
TOTAL OF “DON’T KNOW” ANSWER	17.05		

*Note*. Black answers are correct answers.

**TABLE 3.** Canadian sexual and gender minorities responses to the Literacy of Suicide Scale (LOSS).

Statement	Selected	Selected	Selected
	True	False	Don’t know
People who have thoughts about suicide should not tell others about it.	1.2	96.9	1.9
Most people who suicide are psychotic.	0.6	92.7	6.7
Men are more likely to suicide than women.	90.1	3.0	6.9
Very few people have thoughts about suicide.	1.5	88.0	10.4
Seeing a psychiatrist or psychologist can help prevent someone from suicide.	86.8	6.7	6.5
A suicidal person will always be suicidal and entertain thoughts of suicide.	4.4	80.9	14.7
Not all people who attempt suicide plan their attempt in advance.	80.6	9.9	9.5
If assessed by a psychiatrist, everyone who suicides would be diagnosed as depressed.	6.0	77.1	16.8
Talking about suicide always increases the risk of suicide.	7.4	75.9	16.7
People who talk about suicide rarely kill themselves.	14.3	55.9	29.8
People who want to attempt suicide can change their mind quickly.	49.6	22.0	28.3
There is a strong relationship between alcoholism and suicide.	43.8	20.2	36.0
*LGBTQ people are more likely to attempt suicide than non-LGBTQ people (i.e. heterosexual and cisgender people).	90.1	3.0	6.9
TOTAL OF CORRECT ANSWER	76.5		
TOTAL OF INCORRECT ANSWER	8.1		
TOTAL OF “DON’T KNOW” ANSWER	14.7		

*This item is not part of the validated scale and was not included in the calculation of the total score.

*Note*. Black answers are correct answers.

In terms of suicide literacy, fewer than 50% of the respondents correctly answered the following items: “People who want to attempt suicide can change their mind quickly” (true); 49.8%) and “There is a strong relationship between alcoholism and suicide” (true; 43.8%). Meanwhile, more than 90% of respondents correctly answered the following three items: “People who have thoughts about suicide should not tell others about it” (false; 96.9%), “Most people who suicide are psychotic” (false; 92.7%), and “Men are more likely to die by suicide than women” (true; 90.1%). The same percentage, 90.1, of respondents also answered correctly to the statement “LGBT people are more likely to attempt suicide than non-LGBTQ people” (true; item not included in the tabulation of the scale).

D-LIT and LOSS scores were associated with one another (*r* = .45, *p* < .001) and were significantly associated with several sociodemographic characteristics in linear regressions. D-LIT scores (Table 4) were significantly higher among cisgender women (Beta =0.66, *p* < .001) and nonbinary individuals (Beta = 0.84, *p* < .001) and significantly lower for transgender women (Beta = 0.60, *p* < .040) in comparisons to cisgender men. D-Lit was also significantly higher in individuals aged 30–39 (Beta = 0.77, *p* < .001), and 40–49 (Beta = 0.73, *p* = .007) compared to those under 20. Significantly lower D-LIT scores were found among those whose highest level of education was some college/university (Beta = −0.62, *p* < .001), completed high school education (Beta = −1.71, *p* < .001), and some high school (Beta = −1.59, *p* < .001), compared to those with a university degree, and SGM from Indigenous (Beta = −0.45, *p* = .014) and ethnic minority communities (Beta = −0.45, *p* = .018, compared to White SGM. LOSS scores (Table 5) were significantly lower among those aged 40–49 (Beta = −0.59, *p* = .001) and 50 and over (Beta = −0.90, *p* < .001), compared to those under 20 years old, as well as among SGM whose highest level of education was some college or university (Beta = −0.29, *p* = .001), completed high school education (Beta = −0.72, *p* < .001), or some high school (Beta = −0.66, *p* < .001), when compared to SGM with a university degree. Finally, LOSS scores were significantly lower among SGM from ethnic minority groups (Beta = −0.66, *p* < .001) when compared to White SGM.

**TABLE 4.** Linear regression associations between Depression Literacy Scale (D-lit) score and demographic variables, as measured in a Canadian survey of sexual and gender minorities.

Variables	*B*	SE B	*β*	*t*	*p*
Gender					
Cisgender men	Referent				
Cisgender women	0.66	0.17	.11	4.00	<.001
Transgender men	0.52	0.26	.05	2.05	.040
Transgender women	−0.60	0.35	−.03	−1.72	.085
Nonbinary	0.84	0.20	.11	4.13	<.001
Sexual orientation					
Gay/Lesbian	Referent				
Bisexual	−0.15	0.16	−.02	−0.92	.357
Queer	0.30	0.18	.04	1.65	.100
Asexual	−0.05	0.28	−.00	−0.16	.872
Pansexual	0.18	0.19	.02	0.92	.356
Age					
Under 20	Referent				
20–29	0.27	0.15	.05	1.79	.074
30–39	0.77	0.20	.10	3.85	<.001
40–49	0.73	0.27	.06	3.72	.007
50+	0.17	0.24	.02	0.71	.477
Education					
University degree	Referent				
Some college or university	−0.62	0.14	−.10	−4.56	<.001
High school completed	−1.71	0.20	−.19	−8.60	<.001
Some high school	−1.59	0.28	−.12	−5.65	<.001
Income					
50,000 or more	Referent				
20,000–49,999	−0.01	0.17	−.00	−0.07	.947
Under 20,000	0.30	0.16	.05	1.86	.063
Ethnicity					
White	Referent				
Indigenous	−0.45	0.18	−.03	−2.47	.014
Ethnic minority	−0.45	0.19	−.04	−2.37	.018

**TABLE 5.** Linear regression associations between Literacy of Suicide Scale (LOSS) score and demographic variables, as measured in a Canadian survey of sexual and gender minorities.

Variables	*B*	SE B	*β*	*t*	*p*
Gender					
Cisgender men	Referent				
Cisgender women	0.13	0.11	.03	1.19	.235
Transgender men	0.09	0.16	.01	0.53	.598
Transgender women	−0.33	0.22	−.03	−1.48	.140
Nonbinary	0.09	0.13	.02	0.67	.503
Sexual orientation					
Gay/Lesbian	Referent				
Bisexual	0.03	0.10	.01	0.26	.798
Queer	0.09	0.12	.02	0.80	.426
Asexual	−0.18	0.18	−.02	−1.02	.309
Pansexual	−0.09	0.12	−.02	−0.69	.488
Age					
Under 20	Referent				
20–29	−0.10	0.10	−.03	−1.03	.306
30–39	−0.11	0.13	−.02	−0.88	.377
40–49	−0.59	0.17	−.08	−3.43	.001
50 +	−0.90	0.16	−.14	−5.82	<.001
Education					
University degree	Referent				
Some college or university	−0.29	0.09	−.08	−3.40	.001
Completed high school	−0.72	0.13	−.13	−5.69	<.001
Some high school	−.66	.179	−.08	−3.69	<.001
Income					
50,000 or more	Referent				
20,000–49,999	−0.06	0.11	−.01	−0.60	.553
Under 20,000	0.04	0.10	.01	0.39	.698
Ethnicity					
White	Referent				
Indigenous	−0.01	0.12	−.00	−0.05	.959
Ethnic minority	−0.66	0.12	−.10	−5.43	<.001

## DISCUSSION

The current study is among the first investigation to explore depression and suicide literacy among SGM. Suicide and depression literacy scores were relatively high among the respondents. In comparison, a Canadian online study of the general population that used the same instruments found slightly lower rates of depression literacy (67.1% of correct answers versus 71.2% in the present study) and lower rates of suicide literacy (53.7% of correct answers versus 76.5%; Oliffe et al., 2016). While these results are encouraging for SGM, this population experiences manyfold higher rates of depression and suicide (Hottes et al., 2016; King et al., 2008) and, as such, the present study identified some important gaps in SGM knowledge of depression and suicide that should be of concern to health professionals and policymakers. Specifically, SGM had difficulty answering items related to the treatment of depression. For example, over 60% of SGM were unaware that a clinical psychologist cannot prescribe antidepressants (as is the case in Canada). With regard to suicide literacy, SGM were aware of the increased risk of suicide in their community. Indeed 90.1% correctly answered that SGM individuals were more likely to attempt suicide than non-SGM individuals. A large proportion of SGM were also aware of the need to talk about suicide, that people who die by suicide are not necessarily psychotic, and that in the general population, men are more likely to die by suicide than women. On the other hand, many were unaware of the relationship between alcoholism and suicide, which is of concern in a community at increased risk of alcohol dependence (Arayasirikul, Pomart, Raymond, & Wilson, 2018; Bryan, Kim, & Fredriksen-Goldsen, 2017; Coulter et al., 2015, 2018). Moreover, half of the respondents were unaware that people who are about to enact a suicide plan can change their mind quickly. Addressing this knowledge gap could potentially enable individuals to seek help for a suicidal peer or for themselves.

This study additionally highlights significant inequities in depression and suicide literacy *within* the SGM population. While there was no difference in literacy measures across sexual identities, some significant differences were found across genders. Specifically, cisgender men and transgender women had lower literacy levels when compared to cisgender women. Research among the general population has already described lower literacy among men when compared to women, on average (Oliffe et al., 2016; Wang et al., 2007). The present study, however, is the first to report that this difference also occurs within SGM. Furthermore, this report is the first to measure and compare depression and suicide literacy across transgender and cisgender categories, demonstrating lower average literacy levels among transgender women compared to cisgender women. This difference may be explained, at least in part, by the particular barriers and discrimination transgender women face in accessing health services, such as discrimination from health-care providers and a lack of gender-affirming health services (Grant et al., 2010), as relationships with health providers is an important determinant of health literacy (Paasche-Orlow & Wolf, 2007). While more research is needed to understand the reasons underlying these inequities, our findings nevertheless highlight the importance of accounting for diverse gender identities within mental health literacy research, as well as in the design of mental health literacy interventions for SGM.

The largest differences in literacy were found across educational attainment, where those with lower levels of attainment (high school or uncompleted university) had lower literacy scores in comparison to those who had a university degree. This is not surprising considering the strong links between education, general literacy, and health literacy (Nutbeam, 2000). Nonetheless, the poorer score of SGM with lower educational attainment underscores existing concerns in this area, as SGM with lower educational attainment have been found to be at high risk of depression and suicide (Ferlatte, Salway, Rice, et al., 2019; Ferlatte et al., 2018). These results therefore have implications for the design of interventions to address suicide and depression literacy. Indeed, a recent review of Canadian websites delivering HIV-related information to gay and bisexual men found that most sites required a reading level higher than typically recommended for health literacy interventions (Gilbert et al., 2019). As such, interventions to address depression and suicide literacy should be cognizant of the need for easily accessible information and avoid technical language; a Grade 6 reading level is usually recommended for health literacy interventions (Weiss, 2003). Therefore, health promoters should consider creative modes of delivery and alternatives to reading text such as images, graphics, and videos in the design of interventions (Weiss, 2003).

Alternatives to prose and text in depression and suicide literacy interventions may also benefit another subgroup of SGM who had lower depression and suicide literacy in our study, SGM from ethnic minorities. While this survey did not assess for English and French language skills and did not collect information about respondents’ first language, one potential explanation for lower literacy scores among this subgroup is language barriers. Another hypothesis is that there are differences related to cultural understandings of depression and suicide within some ethnocultural communities in Canada. As such, a consideration for this group is to develop interventions that are culturally appropriate (Resnicow, Baranowski, Ahluwalia, & Braithwaite, 1999), meaning that they include representations of people from diverse ethnocultural communities, attend to cultural understandings of mental health, and discuss specific issues, such as racism, which is linked to mental illness among ethnic minorities (Brondolo, Brady ver Halen, Pencille, Beatty, & Contrada, 2009).

The literature is generally scant when it comes to interventions to promote mental health literacy among SGM. Indeed, most public health interventions to increase the health literacy of SGM have focused on HIV and sexually transmitted infections, and have been primarily designed for gay and bisexual—usually cisgender—men alone (Knight, Karamouzian, Salway, Gilbert, & Shoveller, 2017). One exception is a multimedia campaign aimed at increasing depression literacy among sexual minorities in Switzerland, which was shown to be successful in improving mental health literacy and help-seeking (Jen Wang, Häusermann, Berrut, & Weiss, 2013). In terms of future efforts to promote mental health literacy, web-based interventions are highly promising because SGM tend to have a preference for web-based interventions (versus in-person and offline interventions) (Ferlatte, Salway, Oliffe, et al., 2019; McInroy, McCloskey, Craig, & Eaton, 2019), and web-based interventions have been shown to be acceptable and cost-effective in when promoting health literacy among SGM (Muessig, Nekkanti, Bauermeister, Bull, & Hightow-Weidman, 2015; Rosenberger, Reece, Novak, & Mayer, 2011; Ybarra & Bull, 2007). Similarly, a systematic review of web-based interventions to increase mental health literacy among other populations (i.e., college students, young adults, people with mental illnesses) found that web-based interventions are efficacious, particularly if they had structured programs, delivered evidence-based content, and promoted interactivity and experiential learning (Brijnath, Protheroe, Mahtani, & Antoniades, 2016). Interventions were likely to be successful if tailored to address the unique needs and experiences of a specific populations (Brijnath et al., 2016). As such, we suggest that web-based approaches represent a critical “next step” for interventionists in this area, particularly as web interventions provide an opportunity to tailor approaches to an individual’s specific “profile” (e.g., based on their various intersecting social positionalities, including gender and sexual identities), tend to better capture a user’s attention, contain less redundant information, and overall are more acceptable among users (Lustria et al., 2013).

There are several strengths of this study including the large and diverse sample and the utilization of validated questionnaires (D-LIT and LOSS) to measure depression and suicide literacy. Nonetheless, the findings detailed in this article must be understood in the context of some important limitations. First, the study relied on a non-probability sample and is unlikely to be representative of the SGM Canadian population as evidenced by the gender imbalance of the sample and the high proportion of nonbinary respondents. More so, because our recruitment strategy relied on social media and SGM community groups, we may have missed responses from individuals who are the least connected to SGM communities or have limited access to the Internet. Second, we did not assess the respondents’ history of mental health diagnostics or mental health symptomology, which could be important factors affecting suicide and depression literacy because a history of depression has been associated with depression literacy in a sample of gay men (Wang et al., 2014). Third, while unlikely, it is impossible for us to confirm that respondents did not verify the answers online to the D-LIT and LOSS questionnaires while responding to the survey. Fourth, recognizing depression and suicide symptoms is complex, and as such knowledge of risk factors and symptoms alone may not necessarily indicate that individuals are able to accurately recognize and respond to depression and suicidality in real life contexts. Future studies measuring depression and suicide literacy among SGM could incorporate the use of vignettes, a method that has been employed to study mental health literacy (Jorm et al., 2006; Wei et al., 2015), to assess the depression and suicide literacy skills of SGM in diverse cases and situations. In addition, the D-LIT and LOSS questionnaires rely on outdated definitions of health literacy and only measure the knowledge base of respondents and, as such, it remains unclear how depression and suicide literacy affect health-seeking behaviors. Future studies would benefit from extending the definitions of suicide and depression literacy to include how knowledge is obtained, attitudes including suicide and depression stigma, and help-seeking efficacy because these are components of the evolving definition of mental health literacy (Kutcher, Wei, & Coniglio, 2016).

In conclusion, although an encouragingly high percentage of Canadian SGM show awareness of depression and suicide, the current study results suggest that education efforts are still needed to improve general depression and suicide literacy, particularly among some subgroups of SGM, namely cisgender men, transgender women, Indigenous people, ethnic minorities, and those with lower educational attainment. This is especially relevant based on evidence that mental health literacy can aid early intervention for mental health disorders (Kelly, Jorm, & Wright, 2007). However, the results detailed in this study are preliminary. More work is needed theoretically and empirically to understand how suicide and depression knowledge, attitudes, and skills can improve the capacities of SGM communities to access and use mental health information effectively. Depression and suicide literacy represents only one consideration for improving the mental health of SGM, and more work is also needed to understand how depression and suicide literacy intersect with other determinants of mental health, such as stigma, treatment access, and social support for mental health (Nutbeam, 2000).

## Footnotes

1 The term “sexual and gender minorities” in this article refers to people whose sexual and/or gender identity differ from the majority (i.e,. heterosexual and cisgender) and include, but are not limited to, people who identify as gay, lesbian, bisexual, asexual, pansexual, queer, two-spirit, genderqueer, transgender, and gender nonbinary.

## AUTHOR NOTES

Olivier Ferlatte, Department of Social and Preventative Medicine, School of Public health, University of Montreal, Montreal, Canada; Centre de Recherche en Santé Publique, Montreal, Canada. Travis Salway, Faculty of Health Sciences, Simon Fraser University, Burnaby, Canada; British Columbia Centre for Disease Control, Vancouver, Canada; Centre for Gender and Sexual Health Equity, Vancouver, Canada. John L. Oliffe, School of Nursing, University of British Columbia, Vancouver, Canada. Simon M. Rice, Orygen, The National Centre of Excellence in Youth Mental Health, Melbourne, Australia; Centre for Youth Mental Health, The University of Melbourne, Melbourne, Australia. Mark Gilbert, British Columbia Centre for Disease Control, Vancouver, Canada; School of Population and Public Health, University of British Columbia, Vancouver, Canada. Ingrid Young, Usher Institute of Population Health Sciences and Informatics, University of Edinburgh, Edinburgh, UK. Lisa McDaid, MRC/CSO Social and Public Health Sciences Unit, University of Glasgow, Glasgow, UK. John S. Ogrodniczuk, Department of Psychiatry, University of British Columbia, Vancouver, Canada. Rod Knight, British Columbia Centre on Substance Use, Vancouver, Canada; Department of Medicine, University of British Columbia, Vancouver, Canada.

Correspondence concerning this article should be addressed to Olivier Ferlatte, Department of Social and Preventative Medicine, School of Public health, University of Montreal, C.P. 6128 succursale Centre-Ville, Montréal, QC, H3C 3J7 Canada. Email: olivier.ferlatte@umontreal.ca
